# The long non-coding RNA NEAT1 promotes the progression of human ovarian cancer through targeting miR-214-3p and regulating angiogenesis

**DOI:** 10.1186/s13048-023-01309-9

**Published:** 2023-11-20

**Authors:** Yang Liu, Yan Li, Yanzhi Wu, Yiyue Zhao, Xi Hu, Chunyi Sun

**Affiliations:** 1 Department of Reproduction, the Second Affiliated Hospital of Kunming Medical University, Kunming, 650101 China; 2 Department of Gynecology, the Second Affiliated Hospital of Kunming Medical University, Kunming, 650101 China

**Keywords:** NEAT1, miR-214-3p, Ovarian cancer, Angiogenesis, Metastasis

## Abstract

**Background:**

Angiogenesis and metastasis contributes substantially to the poor outcome of patients with ovarian cancer. We aimed to explore the role and mechanisms of the long non-coding RNA NEAT1 (nuclear enriched abundant transcript 1) in regulating angiogenesis and metastasis of human ovarian cancer. NEAT1 expression in human ovarian cancer tissues and cell lines including SKOV-3 and A2780 was investigated through in situ hybridization. Gene knockdown and overexpressing were achieved through lentivirus infection, transfection of plasmids or microRNA mimics. Cell viability was measured with the cell counting kit-8 assay, while apoptosis was determined by flow cytometry. Cell migration and invasion were evaluated by transwell experiments, and protein expression was determined by western blot assays or immunohistochemistry. Duo-luciferase reporter assay was employed to confirm the interaction between NEAT1 and target microRNA. In vivo tumor growth was evaluated in nude mice with xenografted SKOV-3/A2780 cells, and blood vessel formation in tumor was examined by histological staining.

**Results:**

NEAT1 was highly expressed in ovarian cancer tissues of patients and cell lines. MiR-214-3p was identified as a sponging target of NEAT1, and they antagonizedeach other in a reciprocal manner. NEAT1-overexpressing SKOV-3 and A2780 cells had significantly increased proliferation, reduced apoptosis, and augmented abilities of migration and invasion, while cells with NEAT1-knockdown displayed markedly attenuated traits of malignancies. Additionally, the levels of NEAT1 appeared to be positively correlated with the expression levels of angiogenesis-related molecules, including Semaphorin 4D (Sema4D), Sema4D receptor Plexin B1, T-lymphoma invasion and metastasis-inducing protein-1 (Tiam1), and Rho-like GTPases Rac1/2/3. In the xenograft mouse model, more NEAT1 expression resulted in faster in vivo tumor growth, more blood vessel formation in tumor tissues, as well as higher expression levels of angiogenesis-related molecules and CD31.

**Conclusions:**

NEAT1 promotes angiogenesis and metastasis in human ovarian cancer. NEAT1 and miR-214-3p are promising targets for developing therapeutics to treat human ovarian cancer.

**Supplementary Information:**

The online version contains supplementary material available at 10.1186/s13048-023-01309-9.

## Background

As the most fatal malignancy among gynecologic cancers, ovarian cancer is characterized by rapid growth and intraperitoneal spread [1]. In the USA, approximately 19,880 cases of ovarian cancer are expected to be diagnosed in 2022, and 12,810 of them are expected to result in death [2]. As early detection is difficult, ovarian cancer is frequently diagnosed at an advanced stage when the tumor has metastasized [3]. The high mortality of this cancer is largely explained by the fact that the majority (75%) of patients present at an advanced stage, with widely metastatic disease within the peritoneal cavity [1, 3]. Despite aggressive frontline treatments with surgery and adjuvant chemotherapy, which can result in an 80% response ratio, advanced ovarian cancer in most patients is usually incurable [4, 5]. A 5-year survival rate of less than 25% was observed in women diagnosed with stages III–IV ovarian cancer, largely due to chemo-resistance and metastasis-associated ascites development [6, 7]. Therefore, a thorough understanding of the mechanisms underlying cancer metastasis is required to facilitate the development of more effective treatment approaches in ovarian cancer therapy.

Metastasis is a complex biological process with multiple stages and steps, and the development of a new vascular supply plays a critical role in tumor metastasis [3]. Angiogenesis, the development of new blood vessels from the existing vasculature, is an essential component of solid tumor growth and metastasis [8]. The onset of angiogenesis in tumor tissues marks a phase of rapid proliferation, local invasion, and ultimately metastasis [8], while multiple molecules are found to play coordinated roles in endothelial cell proliferation and assembly of the vessel wall in a variety of abnormal circumstances [9, 10], such as vascular endothelial growth factor (VEGF). In addition, Semaphorin 4D (Sema4D) and its receptor, Plexin-B1, were found to cooperate with VEGF to promote angiogenesis and tumor progression through attracting Plexin-B1-expressing endothelial cells into the tumor to enhance growth and vascularity [11]. Moreover, it was reported that Sema4D alone can elicit a significant angiogenic response to promote tumor growth independent of VEGF in colorectal cancer [12]. Besides the pro-angiogenic effects of Sema4D [13], T-lymphoma invasion and metastasis-inducing protein-1 (Tiam1), was found to be able to promote the angiogenesis in cervical [14] and lung cancer [15]. Tiam1 has been identified a specific activator of Rho-like GTPases Rac1, and the activation Tiam1-Rac signaling plays a crucial role in enhancing invasion and metastasis of various cancers [16, 17]. Thus, these angiogenesis-associated molecules in tumor tissues are good biomarkers for indicating the metastatic states of ovarian cancer cells.

Recently, accumulating studies have revealed that non-coding RNAs (ncRNAs) contribute significantly to the disease progression in cancer patients [18, 19]. Two major types of ncRNAs, long non-coding RNAs (lncRNAs) and microRNAs (miRNAs or miRs), are found to play essential roles in the metastasis of many cancers including ovarian cancer [20, 21]. For example, the lncRNA nuclear enriched abundant transcript 1 (NEAT1) was reported as an oncogene in various malignant tumors, including hepatocellular carcinoma [22], non-small cell lung cancer [23, 24], and breast cancer [25]. Notably, NEAT1 has been reported to promote the proliferation and metastasis of ovarian cancer in multiple reports [26–29]. MicroRNA-214 (miR-214) has been observed to be aberrantly expressed in various cancers and involved in the progression of many malignant tumors [30, 31]. Our previous study demonstrated that miR-214 can directly target Sema4D and inhibited cell proliferation in human ovarian cancer cell line SKOV-3 [32]. However, the connection between NEAT1 and miR-214-3p, and the roles of NEAT1 in angiogenesis of ovarian cancer, have not been intensively elucidated so far.

In this study, we evaluated the expression level of NEAT1 in clinically resected samples from patients with ovarian cancer and two human ovarian cancer cell lines, and identified that a higher level of NEAT1 was associated with the malignancies of ovarian cancer cells. In addition, we also explored the interactions between NEAT1 and miR-214-3p through luciferase reporter assays. Through plasmid-transfection induced NEAT1 overexpression or short hairpin RNA (shRNA)-mediated knockdown of NEAT1 expression, we revealed the critical roles of NEAT1 in promoting the proliferation and angiogenesis of human ovarian cancer cells both under in vitro culturing condition and in a xenograft mouse model. Our study suggested that NEAT1/miR-214-3p pathway is a potential therapeutic strategy to treat the ovarian cancer.

## Results

### The lncRNA NEAT1 promotes cell proliferation and regulates miR-214-3p expression in human ovarian cancer cells

**Fig. 1:**
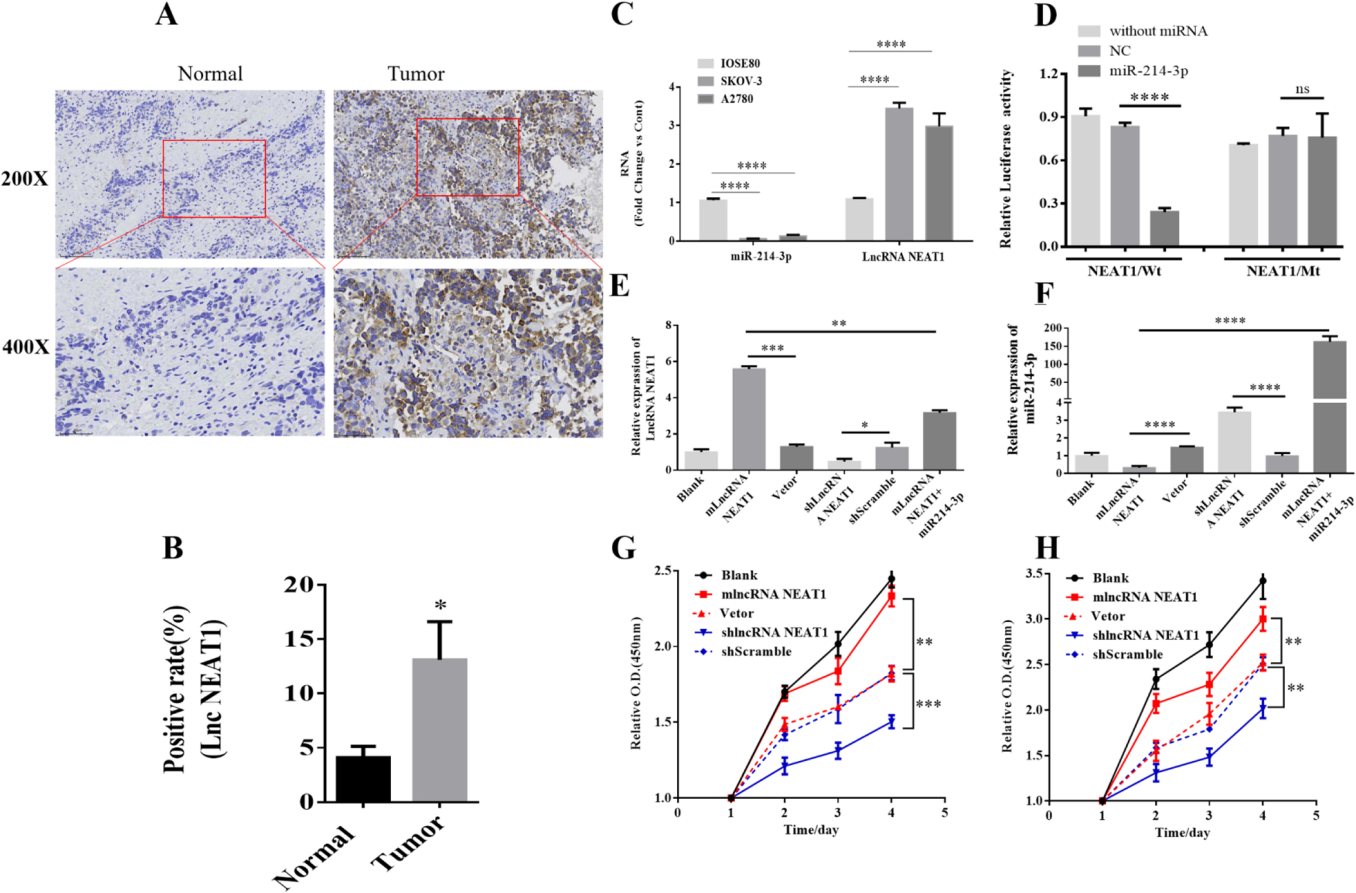
A high expression level of the lncRNA NEAT1 in human ovarian cancer cells suppressed miR-214-3p expression and promoted cell proliferation. **A-B** Human ovarian cancer tissues had more expression of NEAT1 in comparison to the paired non-tumor adjacent normal tissues. **A** Representative images of in situ RNA hybridization using ovarian cancer tissues slide and normal slides. **B** The positive rate of NEAT1-expression cells was summarized. *n* = 3 for each group. **C** The relative RNA levels of miR-214-3p and NEAT1 in human ovarian surface epithelial cell line IOSE80 and human ovarian cancer cell lines SKOV-3 and A2780 were determined by qPCR. **D** The luciferase activity of the reporter vectors was detected in SKOV-3 cells at 48 h after co-transfection of the plasmid expressing wild type or mutant NEAT1 alone (without miRNA), or together with the negative control (NC) mimic, or the miR-214-3p mimic. *n* = 5 for each group. **E–F** The relative levels of NEAT1 (**E**) and miR-214-3p (**F**) in Blank SKOV-3 cells, stable SKOV-3 cells transfected with empty vector or NEAT1-expressing vector, SKOV-3 cells infected with shLncRNA NEAT1- or shScramble-expressing lentivirus, and stable NEAT1-expressing SKOV-3 cells with additional transfection of miR-214-3p mimic were determined by qPCR. **G-H** The proliferation of blank SKOV-3 cells (**G**) and A2780 cells (**H**) or these cells transfected with empty vector or NEAT1-expressing vector (mLncRNA NEAT1), or infected with shLncRNA NEAT1- or shScramble-expressing lentivirus was determined by CCK-8 assays. *P < 0.05, **P < 0.01, ***P < 0.001, ****P < 0.0001, between the indicated groups. Each group was tested with three samples

To explore the potential roles of the lncRNA NEAT1 in the pathogenesis of ovarian cancers, we first evaluated its expression level in cancer samples from patients through in situ RNA hybridization. As shown in Fig. 1A, NEAT1 displayed a significantly higher expression level in ovarian cancer tissues, in comparison to that of the paired normal tissues. The positive rate of NEAT1-expressing tissues in cancer samples was ~ 3 folds of that in the adjacent non-tumor tissues (Fig. 1B). Next, we evaluated the expression levels of NEAT1 and its putative sponging target miR-214-3p in human ovarian surface epithelial cell line IOSE80 and two common human ovarian cancer cell lines SKOV-3 and A2780. We found that SKOV-3 and A2780 cells had over 2.5-fold expression of NEAT1 than IOSE80 cells, while IOSE80 cells showed a markedly higher expression level of miR-214-3p than the two ovarian cancer cell lines (Fig. 1C).

To further confirm the interaction between NEAT1 and miR-214-3p, we constructed luciferase reporter vectors with expression of wild type NEAT1 or mutated NEAT1, and co-transfected these plasmids with either control vehicle or miR-214-3p mimics to SKOV-3 cells. As shown in Fig. 1D, our luciferase assays showed that the luciferase activities after co-transfection of miR-214-3p mimics were significantly decreased when wild type NEAT1 was expressed, but barely changed when mutated NEAT1 was expressed, which implied that NEAT1 is potentially a competing endogenous RNA of miR-214-3p. Moreover, we manipulated the expression levels of these two molecules in SKOV-3 cells through plasmids transfection, lentivirus infection, or miRNA mimics transfection. SKOV-3 cells with higher levels of NEAT1 expression displayed significantly downregulated miR-214 expression, while shRNA-mediated NEAT1 knockdown markedly increased miR-214 level in SKOV-3 cells (Fig. 1E). In addition, additional transfection of miR-214 in NEAT1-overexpression SKOV-3 cells led to prominently downregulated NEAT1 expression (Fig. 1F), suggesting a possible mutual antagonism between these two molecules. Furthermore, we investigated the impacts of upregulated and downregulated NEAT1 expressions on proliferation of human ovarian cancer cells SKOV-3 (Fig. 1G) and A2780 (Fig. 1H). Compared to control vector or shRNA transfected cells, NEAT1-overexpressing cells showed significantly increased proliferation, while NEAT1-silenced cells exhibited considerably decreased proliferation from day 2 to day 4. No significant differences were observed between the blank control and NEAT1-overexpressing cells, or between the control vector and control shRNA transfected cells at any time points (Fig. 1G, H). Therefore, NEAT1 promoted cell proliferation in human ovarian cancer cells.

### NEAT1 helps maintain cell viability and the trait of metastasis in human ovarian cancer cells.

**Fig. 2:**
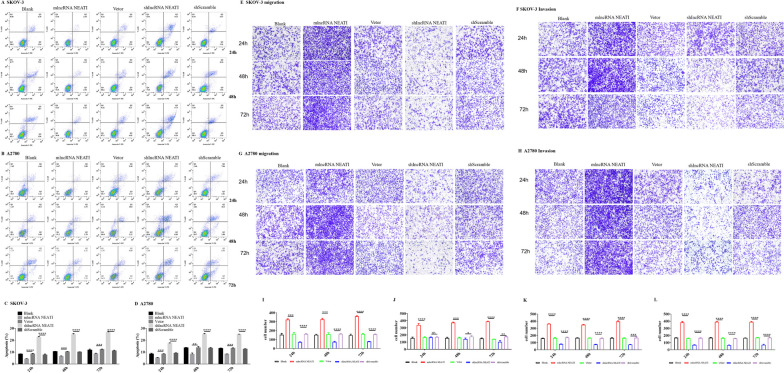
NEAT1 expression in human ovarian cancer cells inhibited apoptosis and promoted cell migration and invasion. **A-D** Apoptosis rates in untreated SKOV-3 or A2780 cells (blank), stable NEAT1-expression cells (mlncRNANEAT1) or control vector-transfected cells (vector), and NEAT1-knockdown cells with infection of shRNA-expressing lentivirus (shlncRNANEAT1) or the corresponding control cells with infection of scramble shRNA-expressing lentivirus (shScramble) were measured by Annexin-V/7-AAD flow cytometrical staining. Representative flow profiles for SKOV-3 (**A**) and A2780 (**B**) cells after culturing for 24 h, 48 h and 72 h are shown. Starting from the bottom left (Q3) and moving in a counterclockwise direction, the quadrants represent live cells (Q3), early apoptotic cells (Q4), late apoptotic cells (Q2), and necrotic cells (Q1), respectively. The percentages of apoptotic cells (Annexin V-positive cells among total cells; **C** for SKOV-3 cells and **D** for A2780 cells) were summarized. **E-H** The abilities of migration (**E**,** G**) and invasion (**F**,** H**) of SKOV-3 (**E**, **F**) or A2780 cells (**G**, **H**) were determined by transwell assays. **I-L** Qualification of migration (**I**, **K**) and invasion (**J**, **L**) of SKOV-3 (**I**, **J**) or A2780 (**K**, **L**) cells. Representative images are shown, and the numbers of migrated or invaded cells were summarized. *n* = 3 for each group; *P < 0.05, **P < 0.01, ***P < 0.001, between the indicated groups

Since NEAT1 demonstrated significantly pro-proliferative effects in human ovarian cancer cells, we further examined the impacts of NEAT1 expression on apoptosis of SKOV-3 cells and A2780 cells, as well as their metastatic behaviors–the cellular traits of many malignant cells. In SKOV-3 cells (Fig. 2A, C), at 24 h, the shlncRNANEAT1 condition had a substantial increase in early apoptosis compared to shScramble. By 48 h, the shlncRNEAT1 condition exhibited a significant increase in both early and late apoptotic events, whereas the mlncRNANEAT1 condition showed reduced apoptotic events compared to its control. By 72 h, all conditions, especially the shlncRNEAT1 condition, displayed a decline in apoptotic events, suggesting potential recovery or other cellular dynamics. In A2780 cells (Fig. 2B, D), similar to the trends seen in SKOV3 cells, the 24 h data showed increased late apoptosis in the shlncRNEAT1 group compared to shScramble, whereas the mlncRNANEAT1 condition exhibited decreased early and late apoptosis compared to the empty vector. By 48 h, the shlncRNEAT1 condition displayed remarkably elevated early apoptotic events compared to shScrambel. Interestingly, by 72 h, while most conditions displayed declined apoptosis, the shlncRNEAT1 group showed a spike in late apoptotic cells. Notably, we observed a peak in apoptosis rate in A2780 cells in the mlncRNANEAT1 condition at 48 h compared to 24 and 72 h. This may be attributed to the peak response of cells to the NEAT-1 expression, suggesting a potential time-sensitive response mechanism.

To investigate the potential biological function of NEAT1 on metastasis, we determined the abilities of in vitro migration and invasion of SKOV-3 and A2780 cells after manipulated expressions of NEAT1. Generally, at different time points, the stable NEAT1-overexpressing cells had significantly more migrated cells in the transwell assays than the stable empty vector-transfected cells, while NEAT1-knockdown cells showed markedly decreased number of migrated cells, which was found in both SKOV-3 cells (Fig. 2E, I) and A2780 cells (Fig. 2G, K). Similarly, a higher level of NEAT1 expression also promoted the invasion of both SKOV-3 cells (Fig. 2F, J) and A2780 cells (Fig. 2H, L) at different time points, except for non-significant changes at 24 h in SKOV-3 cells. Collectively, these results suggest that NEAT1 expression in human ovarian cancer cells reduced apoptosis and promoted their malignant traits of migration and invasion.

### NEAT1 upregulates the expression of angiogenesis-related molecules in human ovarian cancer cells.

**Fig. 3:**
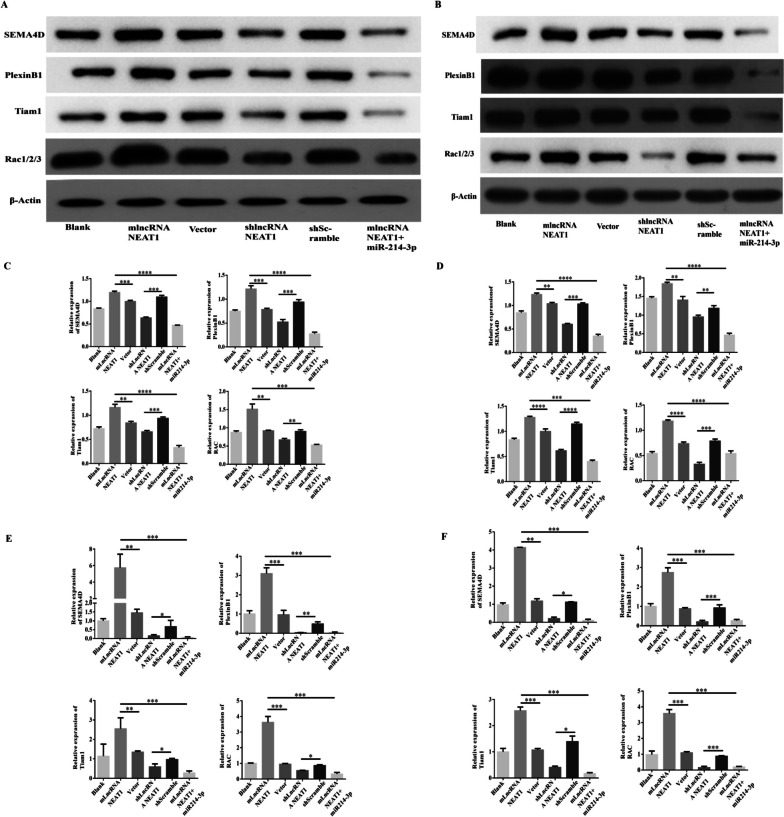
NEAT1 increased the expression of angiogenesis-related molecules in human ovarian cancer cells. **A-D** The protein levels of angiogenesis-related molecules in untreated SKOV-3 or A2780 cells (blank), stable NEAT1-expression cells (mlncRNANEAT1) or control vector-transfected cells (vector), NEAT1-knockdown cells with infection of shRNA-expressing lentivirus (shlncRNANEAT1) or the corresponding control cells with infection of scramble shRNA-expressing lentivirus (shScramble), and stable NEAT1-expression cells with further transfection of miR-214-3p mimics were determined by western blot assays. Representative bands images from SKOV-3 (**A**) and A2780 (**B**) cells are shown, and the relative protein levels of the indicated molecules in SKOV-3 (**C**) and A2780 (**D**) cells were summarized. **E–F** The mRNA levels of the indicated molecules in SKOV-3 (**E**) and A2780 (**F**) cells were quantitated by qPCR. *n* = 3 for each group; *P < 0.05, **P < 0.01, ***P < 0.001, ****P < 0.0001, between the indicated groups

Our previous study demonstrated that miR-214 could directly target Sema4D in human ovarian cancer cells [32]. Considering the interaction between NEAT1 and miR-214-3p and the roles of Sema4D in angiogenesis [11], we further investigated the roles of NEAT1 in regulating the expression levels of angiogenesis-related molecules in SKOV-3 cells and A2789 cells. Blank cells without gene expression manipulation, and the cells after transfection of NEAT-1-expressing plasmid, or infection of NEAT1-specific shRNA-expressing lentivirus, as well as the NEAT1-overexpressing cells upon further transfection of miR-214-3p mimics, were subjected to western blot analysis (Fig. 3A, B) for quantitating the protein levels of Sema4D, Plexin B1, Tiam1, and Rac1/2/3. In both SKOV-3 cells (Fig. 3C) and A2780 cells (Fig. 3D), NEAT1 overexpression showed significantly higher expressions of these angiogenesis-related molecules, while NEAT1 knockdown resulted in decreased expression of these molecules, when compared with the blank and other control groups. In addition, additional miR-214-3p mimics transfection led to considerably downregulated expressions of these molecules in NEAT1-overexpressing SKOV-3 and A2780 cells. Moreover, we also determined the mRNA levels of these molecules in the same groups of SKOV-3 cells (Fig. 3E) and A2780 cells (Fig. 3F) after the same gene expression manipulations. As expected, the same trends in altered expressions of mRNA for these angiogenesis-related molecules were found: the NEAT1 level exhibited a concurrent increase with the mRNA levels of these molecules. Taken together, these results demonstrated that NEAT1 increased the expression of angiogenesis-related molecules in human ovarian cancer cells.

### NEAT1 promotes the growth and angiogenesis of xenografted human ovarian cancer cells in nude mice

**Fig. 4:**
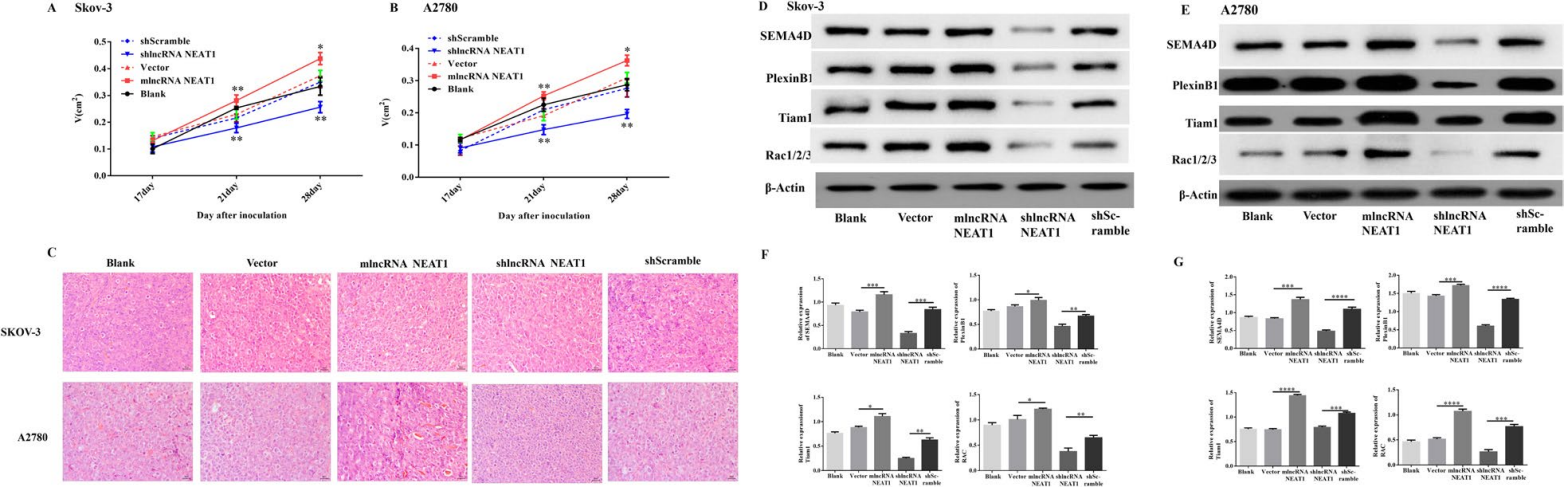
NEAT1 promoted angiogenesis in xenografted human ovarian cancer tissues in nude mice. **A-G** Nude mice were inoculated with untreated SKOV-3 or A2780 cells (blank), stable NEAT1-expression cells (mlncRNANEAT1) or control vector-transfected cells (vector), NEAT1-knockdown cells with infection of shRNA-expressing lentivirus (shlncRNANEAT1) or the corresponding control cells with infection of scramble shRNA-expressing lentivirus (shScramble). Tumor volume was monitored every 7 days starting 17 days after tumor cell inoculation, and mice were euthanized at 30 days after inoculation. **A-B** Tumor growth curves of SKOV-3 (**A**) or A2780 (**B**) cells are shown. **C** Representative HE staining images show the angiogenesis in tumor tissues. Scale bar, 50 µm. **D-E** The protein levels of angiogenesis-related molecules in tumor tissues derived from SKOV-3 (**D**,** F**) or A2780 (**E**,** G**) cells were determined by western blot assays. The representative bands images are shown (**D-E**), and relative protein levels were summarized **(F-G)**. *n* = 5 for each group; *P < 0.05, **P < 0.01, ***P < 0.001, ns, not significant, between the indicated groups

To further consolidate the pro-proliferative and pro-angiogenesis roles of NEAT1, we inoculated SKOV-3 cells and A2780 cells after gene expression manipulations into nude mice, and measured the in vivo tumor growth (Figure S1) and blood vessels formation in tumor tissues. We found that the group of blank cells displayed similar trends in xenograft growth as the control groups with empty vector transfection or scramble shRNA-expressing virus infection. Compared with these control groups, the group with NEAT1-overexpression exhibited markedly enhanced tumor growth, while the group with NEAT1-knockdown showed obviously decreased tumor volumes, which was observed in both SKOV-3 xenograft (Fig. 4A) and A2780 xenograft (Fig. 4B). Additionally, as shown in the results of HE staining (Fig. 4C), the groups of NEAT1-overexpressing displayed evidently more newly formed blood vessels than the blank cell groups and control vector groups, while the shlncRNANEAT1 groups with NEAT1 knockdown had markedly reduced numbers of blood vessels in tumor tissues than the control shScramble group. Moreover, we quantitated the protein levels of angiogenesis-related molecules in the dissected tumor tissues derived from the SKOV-3 xenograft (Fig. 4D) and A2780 xenograft (Fig. 4E) through western blot analysis. Similar as what were observed in in vitro cultures (Fig. 3), tumor tissues with NEAT1-overexpression showed overall more expressions of Sema4D, Plexin B1, Tiam1, and Rac1/2/3 proteins than the control tumor tissues, although no significant differences in protein levels of Plexin B1 from SKOV-3 xenograft (Fig. 4F) and Rac1/2/3 from both SKOV-3 xenograft (Fig. 4F) and A2780 xenograft (Fig. 4G) were identified. However, compared with the control shScramble group, the shlncRNANEAT1 group with silenced NEAT1 had significantly decreased protein levels of all the four molecules in both xenografts (Fig. 4F, G).

**Fig. 5:**
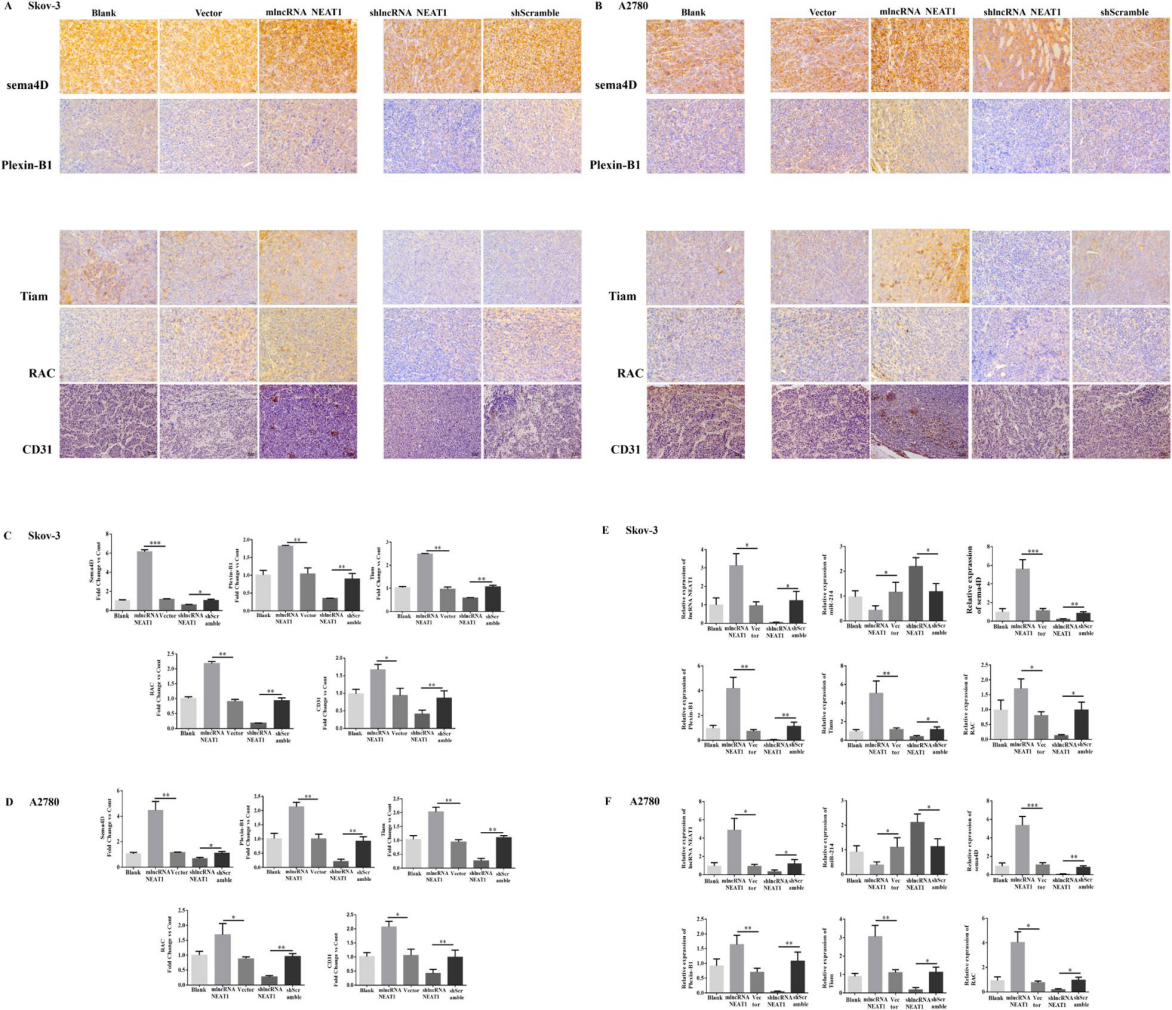
NEAT1 increased the expressions of angiogenesis-related molecules in xenografted human ovarian cancer tissues in nude mice. **A-B** Representative IHC images show the protein expressions of angiogenesis-related molecules in tumor tissues derived from SKOV-3 (**A**) or A2780 (**B**) cells. **C-D** The relative protein levels of the indicated angiogenesis-related molecules were summarized. The IHC staining intensities were normalized to the blank SKOV-3 (**C**) or A2780 (**D**) cells to calculate the fold-change of protein levels. **E–F** The RNA expression levels of NEAT1, miR-214-3p, and angiogenesis-related molecules in tumor tissues derived from SKOV-3 (**E**) or A2780 (**F**) cells were determined by qPCR. *n* = 5 for each group; *P < 0.05, **P < 0.01, between the indicated groups

To further confirm the in vivo pro-angiogenesis roles of NEAT1, we measured the protein levels of four angiogenesis-related molecules as well as CD31, a sensitive and specific marker for vascular differentiation [33], through IHC analysis (Fig. 5A, B). Quantitative analysis of IHC images showed that the NEAT1-overexpressing groups had significantly more protein expressions of all the five molecules in comparison to the control vector groups, while the shlncRNANEAT1 groups with NEAT1 knockdown displayed markedly downregulated expressions of these proteins. In addition, the same trends on the alterations of protein levels were identified in tumor tissues from both SKOV-3 xenograft (Fig. 5C) and A2780 xenograft (Fig. 5D). Furthermore, we performed qPCR analysis to double confirm that these alterations were related to the NEAT1-miR-214-3p axis. As expected, compared with the control groups, the mLncRNANEAT1 group showed a higher NEAT1 level while the shlncRNANEAT1 group had a much lower NEAT1 level. Notably, an opposite alteration in miR-214-3p expression was found in these cells. Additionally, the same trends on the alterations in the mRNA expression levels of the four angiogenesis-related molecules were found to be similar to the alterations in their protein expression levels, in tumor tissues from both SKOV-3 xenograft (Fig. 5E) and A2780 xenograft (Fig. 5F). Thus, the data presented herein clearly demonstrated that NEAT1 promoted in vivo angiogenesis in xenografted human ovarian cancer tissues.

## Discussion

Metastasis is one of the most important reasons that lead to the poor outcome of patients with ovarian cancer, and angiogenesis is an essential component of the metastatic pathway [34]. LncRNA-miRNA interaction has been largely investigated in recent studies in order to reveal the molecular mechanisms involved in regulating cancer progressions [35]. Here, we found that the lncRNA NEAT1 is significantly up-regulated in the ovarian cancer cell lines and clinical cancer tissues in comparison to the corresponding non-tumor cells and tissues. NEAT1 knockdown in SKOV-3 and A2780 cells markedly inhibited cell proliferation, increased apoptosis, and decreased the abilities of cell migration and invasion. Notably, the expression level of NEAT1 positively correlated with the expression levels of angiogenesis-related molecules Sema4D, Plexin B1, Tiam1, and Rac1/2/3 in SKOV-3 and A2780 cells with differentiated expression levels of NEAT1.1 Their correlations were observed in both in vitro cell cultures and xenografted tumor tissues from nude mice. Moreover, a luciferase assay demonstrated a direct interaction between NEAT1 and miR-214-3p. Our study identifies an NEAT1/miR-214-3p pathway in regulating angiogenesis and cancer metastasis, and suggests that the approaches targeting the molecules in this pathway can be utilized to treat ovarian cancer.

We found that NEAT1 was highly expressed in human ovarian cancer cells. The expression level of NEAT1 appeared to be positively correlated with the viability of SKOV-3 and A2780 cells, which suggests that NEAT1 might be a possible biomarker in predicting overall and disease-free survival outcomes of patients with ovarian cancer. Consistent with our findings, Chen et al.reported that NEAT1 was upregulated in ovarian cancer tissues compared with the corresponding adjacent non-neoplastic tissues, and the NEAT1 expression level was an independent factor in predicting the overall survival of ovarian cancer patients [36]. In addition, another report also found that upregulated NEAT1 were found in most ovarian tissues (53/67, 79.10%) when compared with para-tumor tissue specimens [26], and highly-expressed NEAT1 was closely correlated with a shorter survival rate, a poor differentiated degree, a bigger tumor size, an advanced FIGO stage and significant peritoneal metastasis [26]. Thus, NEAT1 is a promising biomarker for predicting the prognosis of patients with ovarian cancer. Crucially, our research also indicates that NEAT1 acts as an oncogenic lncRNA, as its overexpression significantly enhanced cell proliferation and increased the viability of SKOV-3 and A2780 cells. Therefore, NEAT1 expression in tumor tissues might be associated with the advanced development of ovarian cancer, and these results further strengthen that NEAT1 represents an important biomarker in the prediction of ovarian cancer progression. Furthermore, our study demonstrated that NEAT1 silencing remarkably weakened the abilities of migration, invasion of SKOV-3 cells and A2780 cells, and downregulated the angiogenesis potential in cancer cells both in vitro and in vivo, which further substantiate the rationale of targeted inhibition of NEAT1 to prevent or mitigate metastasis for ovarian cancer therapy. Collectively, our findings strengthen the role of NEAT1 as a biomarker for ovarian cancer prognosis and progression, with therapeutic implications.

The tumor-promoting roles of NEAT1 in regulating proliferation, migration, and invasion of human ovarian cancer cells have been documented in multiple studies. While we specifically investigated the SKOV-3 and A2780 cell lines, other research groups employing different cell lines, such as ES-2 cells [29], OVCAR3 cells [28], HO8910 cells [27], have obtained the same conclusion showing the oncogenic roles of NEAT1 expression in enhancing the malignant traits of human ovarian cancer cells. In addition to employing CCK-8, transwell migration and invasion assays, our study utilized wound healing, cell colony formation ability, and in vivo xenograft animal models to further substantiate the role of NEAT1 in promoting ovarian cancer progression. In this study, we quantitated the expression levels of angiogenesis-related molecules Sema4D, Plexin B1, Tiam1, and Rac1/2/3, and found a positive correlation between the level of NEAT1 and the levels of these molecules. Although the in vitro data could not clearly tell a more formation of blood vessels in culturing cancer cells upon NEAT1 overexpression, our in vivo xenograft model did show more signs of blood vasculature inside of the tumor tissues with a higher expression level of NEAT1, possibly due to the recruitment of mouse endothelial cells during blood vessels formation. In line with our finding on the pro-angiogenesis role of NEAT1, a report from Yuan et al.demonstrated that human umbilical vein endothelial cells (HUVECs) had more expressions of VEGF, angiogenin 1 (Ang-1) and matrix metalloproteinase 2 (MMP2), after co-culturing with NEAT1-overexpressing SKOV-3 cells, in comparison to the condition of co-culturing with control SKOV-3 cells [37]. Collectively, these consistent results with indirect or direct evidence again consolidate the oncogenic roles of NEAT1 in promoting angiogenesis in human ovarian cancer cells.

LncRNAs have the capacity to interact with proteins, DNAs or RNAs to act versatile functions [38, 39]. Many studies have revealed that some lncRNAs can act as miRNA sponges by competitively interacting with miRNAs to reduce miRNA availability to their target mRNAs [38, 39]. Using bioinformatics databases, we found that miR-214-3p was one of the target microRNAs of NEAT1. We validated this interaction through a dual luciferase reporter assay, confirming miR-214-3p as a target of NEAT1. Consistent with our results in human ovarian cancer, NEAT1 and miR-214 were also reported to have a reciprocal repression correlation in expressions in human thyroid carcinoma [40]. Evidently, miR-214-3p appears to be a tumor suppressor factor, as it was reported to inhibit the disease progression of ovarian cancer [32, 41], lung squamous cell cancer [42], and colorectal cancer [43]. However, a recent study showed that miR-214-3p was highly expressed in tissues and exosomes derived from human epithelial ovarian cancer with high malignancy, and inhibition of miR-214-3p can help prevent epithelial ovarian cancer malignancy [44]. These discrepancies might be due to the disparity of different patients’ cohorts or the in vitro cell model: OV90 and ES2 cells [44], but not SKOV-3 and A2780 cells, were used for experiments. Interestingly, we found that transfection of miR-214-3p mimics in NEAT1-overexpressing SKOV-3 cells downregulated NEAT1 expression, suggesting that NEAT1 and miR-214-3p inhibited each other in a reciprocal manner. It was reported that the RNA-binding protein (RBP) LIN28B bound to and stabilized NEAT1 in high-grade serous ovarian carcinoma (HGSOC) [28]. Whether manipulated ectopic miR-214-3p expression could impact the abundance of LIN28B or other RBPs to regulate NEAT1 expression, remains to be further investigated. Multiple putative miRNA targets of NEAT1 and the downstream proteins have been proposed to contribute to the oncogenic roles of NEAT1 in ovarian cancer, such as the NEAT1/miR-1312/TJP3 [29], NEAT1/miR-506 [28], NEAT1/let-7 g/MEST/ATGL [27], NEAT1/miR‐382‐3p/ROCK1 [26], and NEAT1/miR-365/FGF9 [37] axes. Therefore, it is plausible that rather than a single pathway, a complexed network of multiple pathways is involved in executing the oncogenic function of NEAT1 in ovarian cancer cells.

This study has some limitations. To the best of our knowledge, this is the first report on revealing the pivotal role of the NEAT1/miR-214-3p axis in regulating angiogenesis and metastasis of human ovarian cancer. However, only two cell lines SKOV-3 and A2780 were chosen for in vitro and in vivo experiments, and more cancer cell lines with differed malignancies might be able to represent the ovarian cancer at different disease stages more faithfully. Another limitation is its sample size of clinical tissues and single data source when evaluating the expression levels of NEAT1 in tumor and non-tumor tissues. The clinical samples from a large cohort can help evaluate the reliability of NEAT1 as a biomarker for ovarian cancer, which might be also employed to predict the prognosis of patients receiving chemotherapy, targeted therapy, and immunotherapy. In addition, more work on validating the therapeutic effects of in vivo knockdown of NEAT1, as well as the delivery of miR-214-3p mimics, in xenograft animal models is warranted to substantiate the potential of targeting the NEAT1/miR-214-3p axis to treat patients with ovarian cancer. Furthermore, it is worth evaluating the efficacy of the targeted NEAT1 inhibition in combination with other therapies (including chemotherapy, targeted therapy, and immunotherapy) for treating ovarian cancer. For example, through suppressing angiogenesis, targeted NEAT1 inhibition potentially enhances immune cell infiltration by promoting vascular normalization, thus improving the efficacy of immunotherapy.

## Conclusions

In summary, we showed that a higher expression level of the lncRNA NEAT1 promoted cell proliferation, reduced apoptosis, enhanced the abilities of migration and invasion, and augmented the potential of angiogenesis in human ovarian cancer cell lines SKOV-3 and A2780, while NEAT1 knockdown remarkably attenuated these malignant traits. In addition, miR-214-3p was identified as a sponging target of NEAT1, and they antagonize each other in a reciprocal manner. These findings provide new insights into the mechanisms underlying cancer metastasis, and suggest that the NEAT1/miR-214-3p pathway can be potentially targeted to develop therapeutics to treat human ovarian cancer.

## Methods

### Patient samples and in situ hybridization

Ovarian cancer tissue microarray slides were obtained from patients recruited in the Second Affiliated Hospital of Kunming Medical University (Kunming, China), and included 3 tumor samples (epithelial ovarian cancer, Stage III/IV) and normal tissue samples (non-tumor adjacent tissue of ovarian). Histological diagnoses of the ovarian cancer samples were provided along with the tissue microarray slides. The digoxin (DIG)-labeled antisense and sense RNA probes for the full lengths of NEAT1 were used to detect NEAT1 expression in the tissue slides, as previously reported [45]. All procedures were conducted following a protocol approved by the Ethics Committee of the Second Affiliated Hospital of Kunming Medical University (RE-PJ-2021–01), with written informed consent.

### Cell culture

The immortalized human ovarian surface epithelial cell line IOSE80 and human ovarian cancer cell line SKOV-3 were purchased from the American Type Culture Collection (Manassas, VA, USA). The human ovarian cancer cell line A2780 was purchased from Kunming Institute of Zoology, Chinese Academy of Sciences (Kunming, China). All the cell lines were tested as mycoplasma contamination free, and authenticated by STR (Short Tandem Repeat) DNA profiling analysis. Cells were maintained in Dulbecco's Modified Eagle's Medium (DMEM; Hyclone™, USA) supplemented with 10% fetal bovine serum (FBS; Gibco, Gaithersburg, MD, USA), 100 units/mL of penicillin and 100 µg/mL of streptomycin (Gibco) at 37 °C in a humidified chamber with 5% CO_2_.

### Gene overexpression and knockdown

The complementary DNA (cDNA) of NEAT1 was cloned into the lentivirus vector pLv201 (Invitrogen, Waltham, MA, USA), and the empty vector plasmid or NEAT1-expressing plasmid was used for transfection of human ovarian cancer cells. For cell transfection, SKOV-3 or A2780 cells were seeded into 6-well or 12-well plates and transfected with the indicated plasmids or miRNA mimics mixed with Lipofectamine 3000 reagent (Thermo Fisher Scientific, Waltham, MA, USA) following the manufacturer's instructions. Stable cell lines were obtained after screening using 2 μg/mL puromycin (Sigma-Aldrich, St. Louis, MO, USA). The miRNA mimics were designed and synthesized by Shanghai GenePharma Co., Ltd (Shanghai, China).

For NEAT1 knockdown, cells were infected with shRNA-expressing lentivirus particles, and the cells infected with scramble shRNA-expressing lentivirus were used as negative controls. The sequences of the shRNA are as follows: shLncRNA NEAT1, 5'-CTGTGAAATGCGGGTAAATGAATG-3'; shScramble, 5'-GAAGTGGCTAGACCTGACGCTAGG-3'. Virus packaging was conducted by co-transfection of lentiviral plasmids and the helper plasmids into 293 T cells (originally obtained from ATCC), and 48 h later, the viruses were harvested to infect the SKOV-3 or A2780 cells in logarithmic growth phase with polybrene at a multiplicity of Infection (MOI) of 20.

### Cell viability

Control SKOV-3 or A2780 cells without transfection, stable SKOV-3 or A2780 cells transfected with empty vector or NEAT1-expressing vector, and SKOV-3 or A2780 cells expressing shNEAT1 RNA or scramble shRNA were seeded in 96 well-plates at the density of 5 × 10^3^/well. The cell counting kit-8 assay (Sigma-Aldrich, St. Louis, MO, USA) was performed according to the manufacturer’s instructions. In brief, 10μL of CCK-8 solution was added to each well of different plates on Day 1, Day 2, Day 3, and Day 4, respectively, and the cells were incubated with the solution for another 1.5 h. Optical density (OD) values were measured at 450 nm using a microplate reader (BioTek, Winooski, VT, USA) to indicate the relative cell viability. The assays were carried out with triplicated samples per group, and the value of Day 1 in the control group was employed for normalization.

### Cell migration and invasion

For cell migration assays, control SKOV-3 or A2780 cells without transfection, stable SKOV-3 or A2780 cells transfected with empty vector or NEAT1-expressing vector, and SKOV-3 or A2780 cells expressing shNEAT1 RNA or scramble shRNA were added into the upper chamber of the Transwell plate with 8 μm pore size (Sigma-Aldrich). For cell invasion assays, the upper chamber of the insert was pre-coated with Matrigel (ECM gel, Sigma-Aldrich). Cells were seeded in serum-free culture medium, and the medium containing 10% FBS in the lower chamber was served as chemoattractant. After incubation for 24 h, 48 h and 72 h, the cells that did not invade through the pores were carefully wiped out with a cotton swab. Then, the inserts with migrated or invaded cells were fixed with 4% paraformaldehyde, and stained with 0.5% crystal violet for 20 min. Cells in five randomly selected visual fields per chamber were counted using an inverted microscope (Motic, Wetzlar, Germany) at 200 × magnification. Cell images were taken using the Moticam (Motic).

### Flow cytometry

Apoptosis was determined using the Annexin V-phycoerythrin (PE) apoptosis detection kit (BD Biosciences, Franklin Lakes, NJ, USA) following the manufacturer’s protocol. Briefly, after washing with phosphate-buffered saline (PBS) and the binding buffer for one time each, cells were stained with Annexin V-PE/7-Aminoactinomycin D (7-AAD) for 20 min at room temperature in dark. After washing with the binding buffer once, the labeled cells were detected immediately by a flow cytometer (Beckman Coulter, Brea, CA, USA). Data were analyzed by the FlowJo software (FlowJo, BD Biosciences).

### Duo-luciferase reporter assay

The binding site of miR-214-3p and the lncRNA NEAT1 was predicted using the online tools LncBase Module (http://diana.imis.athena-innovation.gr/DianaTools/index.php?r=lncBase/index) and SPMLMI [46]. The predicted miR-214 binding site region of NEAT1 (5'- TGGCTAGCTCAGGGCTTCAG-3') and the corresponding mutated region (5'- ACCGATCGAGTCCCGAAGTC-3') were cloned into a Gaussia luciferase (Gluc) and secreted alkaline phosphatase (SEAP)-expressing vector pMT05-01 (Invitrogen, Waltham, MA, USA). SKOV-3 cells were co-transfected with the indicated vectors and the negative control mimic, or the miR-214-3p mimic. At 48 h after transfection, the culture supernatant and cells were harvested and subjected to luciferase activity analysis using a Dual-Luciferase Reporter Assay System (GeneCopoeia, Rockville, MD, USA) following the manufacturer’s instructions. The relative luciferase activity was quantitated according to the ratios of Gluc to SEAP.

### Histology and immunohistochemistry

Tumor tissues immersed in 10% formalin solution were fixed at 4 °C for 24 h, and were then dehydrated with different grades of ethanol. The transparent tissue was embedded into paraffin, cut into 5 µm slices with a microtome, and stained with hematoxylin and eosin (H&E). The histopathological changes of the tissue sections were observed using a Zeiss Axio Lab A1 microscope (Gottingen, Germany), and images were taken with the magnification of 200 × .

For immunohistochemistry staining, the paraffin embedded tumor tissue specimens were subjected to deparaffinization in xylene and rehydration through a series of descending grades of alcohol solutions. Sections were incubated within 3% hydrogen peroxide to block endogenous peroxidase activity followed by microwaved in citrate buffer for antigen retrieval. Nonspecific staining was blocked by incubation with 10% normal goat serum in PBS for 30 min. Sections were incubated with monoclonal mouse antibodies against human SEMA4D (1:100 dilution), Plexin-B1 (1:50 dilution), Tiam1 (1:100 dilution), or RAC (1:100 dilution), CD31 (1:200 dilution) (Abcam, Cambridge, UK) at 4 °C overnight, followed by PBS washes and a further incubation with Horseradish peroxidase (HRP)-labeled anti-mouse immunoglobulin G (IgG)(H + L) (Zsbio, Beijing, China) at room temperature for 2 h. Thereafter, the sections were stained with diami-nobenzidine (DAB) using the 3,3’-diaminobenzidine chromogenic substrate kit (Zsbio, Beijing, China). The positively stained cells in randomly selected 6 visual fields of each group were counted. The positivity percentages were determined as follows: (number of positive cells)/(total number of cells) × 100, and normalized to the group of mice inoculated with un-transfected SKOV-3 cells.

### Real-time quantitative PCR (qPCR)

Total RNA was isolated using TRIzol reagent (Invitrogen, Carlsbad, CA, USA) following the manufacturer’s instructions. Total RNA (1 μg each sample) was used to synthesize cDNA utilizing the all-in-one first-strand cDNA synthesis kit (GeneCopoeia, China). Quantitative PCR for miRNA and mRNA were performed using a standard protocol from the SYBR green master mix (GeneCopoeia) on a 7900HT Real-Time PCR System (Applied Biosystems, Foster City, CA, USA). Relative quantification was determined by normalization to beta-actin (for mRNA or lncRNA) or U6 (for miRNA). The sequences of primers for qPCR analysis are listed in Table 1. PCR primers were synthesized by Sangon Biotech Co. Ltd (Shanghai, China). The PCR reaction protocol consisted of two steps: step one, initial denaturation for 30 s at 95 ℃; step two, denaturation for 5 s at 95 ℃, annealing and extension for 31 s at 60 ℃ and fluorescence signal acquisition. The reactions had a total of 40 cycles, and ended with a melting curve which consisted of 15 s at 95 ℃, 1 min at 60 ℃, 15 s at 95 ℃ and 15 s at 60 ℃. The experiments were repeated for 3 times and each sample was run in triplicates. PCR product specificity was confirmed by melting curve analysis. Gene expression levels were calculated with the 2^−ΔΔCT^ method [47].

**Table 1:** The sequences of qPCR primers in this study

Gene	Primer sequence (5’-3’)
β-actin	F, CCAGGGCGTTATGGTAGGCA; R, TTCCATATCGTCCCAGTTGGT
lncRNA NEAT1	F, TGGCTAGCTCAGGGCTTCAG; R, TCTCCTTGCCAAGCTTCCTTC
U6	F, CTCGCTTCGGCAGCACATATACT; R, ACGCTTCACGAATTTGCGTGTC
miR-214	F, ACAGCAGGCACAGACAGGCAGU; R, UGCCUGUCUGUGCCUGCUGUUU
Sema4D	F, GAAAAGGGGAAATCAAAACA; R, CGTCAGCAAACACGAAACTA
PlexinB1	F, ACCAAGTGGATAAGGG R, ATTCAAGGTCAGGGGA
Tiam1	F, TTTCGTTTCCGCTGTTATTT R, CACTTCTTTCTCCCTCTTGC
Rac1/2/3	F, CCGAGGTGCTGGAGGACAATGAC R, GCCGAGTAGGAGAACTGGGGGAA

*F* Forward, *R* Reverse

### Western blot

After washing with cold PBS, SKOV3 or A2780 cells or these cell-derived xenograft samples were lysed in radioimmunoprecipitation assay lysis buffer (RIPA; Sigma-Aldrich) containing a protease and phosphatase inhibitor cocktail on ice for 30 min. After centrifugation at 16,000 g for 10 min at 4˚C, proteins in the supernatants were quantified and equal amounts of total proteins were loaded. Samples were separated by 10% SDS-PAGE, transferred to polyvinylidene difluoride (PVDF) membranes, and incubated with indicated primary antibodies at room temperature for 1 h. These antibodies were as follows: anti-SEMA4D antibody (1:1,000 dilution; Affinity Biosciences, USA), anti-VEGF (1:1000; Abcam, Cambridge, UK), anti-XBP1 (1:1000; Abcam), and anti-β-actin (1:5000; Abcam). The secondary horseradish peroxidase (HRP)-conjugated anti-rabbit IgG (1:10,000; TransGen Biotech, Inc., Beijing China) or anti-mouse IgG (1:10,000; TransGen Biotech, Inc.) antibodies were used. Proteins of interest were visualized using enhanced chemiluminescence kit (EMD Millipore, Burlington, MA, USA). The band intensities were quantified by densitometry using the ImageJ software (National Institutes of Health, Bethesda, MD, USA). Western blots of all the experiments were repeated at least 3 times and one representative blotting result is shown for each experiment.

### Xenograft animal model

Six-eight-week-old female BALB/c nude mice were purchased from Hunan SJA Laboratory Animal Co., Ltd (Changsha, China), and housed at the specific pathogen-free (SPF) facility at the Animal Center of Kunming Medical University (Kunming, China). Mice were maintained at room temperature (22 ± 1 ˚C) with a 12/12 h light/dark cycle and access to food and water ad libitum. Control SKOV-3 or A2780 cells without transfection, stable SKOV-3 or A2780 cells transfected with empty vector or NEAT1-expressing vector, and SKOV-3 or A2780 cells expressing shNEAT1 RNA or scramble shRNA were injected subcutaneously into the right inguen flank of each mouse (1 × 10^7^ cells/mouse) to establish the xenograft model (*n* = 5/group). Tumor volume (V) was monitored every 7 days by measuring the tumor diameters starting seventeen days after tumor inoculation, and calculated with the following formula: V = ab^2^/2, where *a* is the long diameter, while *b* is the short diameter. Thirty days after tumor inoculation, mice were euthanized and tumor tissues were frozen at -80C for further analyses. Animal experiments were conducted in accordance with the Declaration of Helsinki and all procedures involving experimental animals were approved by the University Committee on the Use and Care of Animals (UCUCA) at the Kunming Medical University.

### Statistics

Statistical analysis was performed with SPSS (Version 22.0; IBM Corporation, Armonk, NY, USA). All the parameters underwent normality tests. Data were expressed as the mean ± standard deviation. Group differences were analyzed using one-way ANOVA, followed by Bonferroni post-hoc tests for pairwise comparisons. Paired *t*-tests were performed for group comparisons, with *P* < 0.05 indicating statistical significance.

### Supplementary Information

**Additional file 1.**

## Data Availability

All data generated or analysed during this study are included in this published article.

## Abbreviations

Sema4D: Semaphorin 4D
Tiam1: T-lymphoma invasion and metastasis-inducing protein-1
VEGF: Vascular endothelial growth factor
ncRNAs: Non-coding RNAs
lncRNAs: Long non-coding RNAs
shRNA: Short hairpin RNA
OD: Optical density
PBS: Phosphate-buffered saline
PVDF: Polyvinylidene difluoride
HRP: Horseradish peroxidase
SPF: Specific pathogen-free
